# Effect of Walnut Meal Peptides on Hyperlipidemia and Hepatic Lipid Metabolism in Rats Fed a High-Fat Diet

**DOI:** 10.3390/nu13051410

**Published:** 2021-04-22

**Authors:** Xiao-Yue Yang, Di-Ying Zhong, Guo-Liang Wang, Run-Guang Zhang, You-Lin Zhang

**Affiliations:** College of Food Engineering and Nutritional Science, Shaanxi Normal University, Xi’an 710119, China; y550684149@163.com (X.-Y.Y.); zhongdiying@163.com (D.-Y.Z.); guoliangw@snnu.edu.cn (G.-L.W.); sunshine@snnu.edu.cn (R.-G.Z.)

**Keywords:** walnut meal, peptides, hyperlipidemia, high-fat diet

## Abstract

As a natural active substance that can effectively improve blood lipid balance in the body, hypolipidemic active peptides have attracted the attention of scholars. In this study, the effect of walnut meal peptides (WMP) on lipid metabolism was investigated in rats fed a high-fat diet (HFD). The experimental results show that feeding walnut meal peptides counteracted the high-fat diet-induced increase in body, liver and epididymal fat weight, and reduce the serum concentrations of total cholesterol, triglycerides, and LDL-cholesterol and hepatic cholesterol and triglyceride content. Walnut meal peptides also resulted in increased HDL-cholesterol while reducing the atherosclerosis index (AI). Additionally, the stained pathological sections of the liver showed that the walnut meal peptides reduced hepatic steatosis and damage caused by HFD. Furthermore, walnut meal peptide supplementation was associated with normalization of elevated apolipoprotein (Apo)-B and reduced Apo-A1 induced by the high-fat diet and with favorable changes in the expression of genes related to lipid metabolism (LCAT, CYP7A1, HMGR, FAS). The results indicate that walnut meal peptides can effectively prevent the harmful effects of a high-fat diet on body weight, lipid metabolism and liver fat content in rats, and provide, and provide a reference for the further development of walnut meal functional foods.

## 1. Introduction

Obesity and metabolic syndrome are becoming more prevalent in developing countries as economic development increases, mainly due to accelerated urbanization, nutritional transformation and reduced physical activity, as well as genetic factors [1,2]. Not only in adults, but obesity has become increasingly prominent among children, with the prevalence of childhood obesity increasing by approximately 5% per decade over the past 50 years [3]. The accumulation of fat caused by obesity will lead to dyslipidemia and metabolic syndrome. Hyperlipidemia is a risk factor associated with atherosclerosis and subsequent cardiovascular diseases [4]. The formation of atherosclerosis is closely related to lipids and lipid-containing substances in the blood, of which cholesterol has the greatest impact [5]. The current anti-obesity drugs, including orlistat, locasserin, and sustained-release tablets of phentermine/topiramate, naltrexone/bupropion exhibit some degree of efficacy at weight loss [6,7]. However, these anti-obesity drugs also show side effects, such as gastrointestinal diseases, weakness, mental disorders, cardiovascular diseases, and so on [8]. Therefore, many experts expect to obtain hypolipidemic active factors from natural products to intervene in obesity and its related complications in addition to direct drug treatment.

At present, natural products of polyphenols, polysaccharides and peptides are widely studied. Catechins in tea [9,10] and flavonoids in citrus [11,12] can influence lipoprotein metabolism and improve hyperlipidemia; tea polysaccharides [13], and mushroom polysaccharides [14] can reduce blood lipids; dietary protein and peptides derived from vegetables and animals can reduce cholesterol [15]. Phytogenic peptides have been extensively studied in the prevention of obesity in recent years. For example, soy peptides have been widely proven to reduce cholesterol and triglycerides, and inhibit fat synthesis and storage [16,17,18]. In addition to soybean peptides, lower weight gain and hepatic lipid content in hamsters fed high-fat diet were observed when supplemented with brown rice protein hydrolysates [19]; bamboo shoots have shown the effect of lowering cholesterol and regulating lipid metabolism [20]; peptides from cowpea inhibit cholesterol synthesis and its solubilization into micelles [21].

Walnuts (*Juglans regia L.*) have high medicinal value since ancient times. They have several beneficial effects including reducing inflammation, improving endothelial function, and anti-cancer [22,23,24]. Moreover, the consumption of nuts in general and walnuts in particular has been associated with reduced rates of cardiovascular disease [25]. Dietary walnuts can reduce liver triglyceride content in high-fat mice by regulating liver fatty acid metabolism and fatty tissue inflammation [26]. Walnut meal, as a by-product of the cold-squeezed walnut kernel oil, is rich in nutrients and high in protein. Bioactive peptides obtained by enzymatic hydrolysis of nut proteins have better functions than proteins [27]. In recent years, research on walnut peptides focused on ACE inhibitory peptides that decrease the blood pressure of hypertensive patients [28,29]. In addition, walnut peptides have been shown to reduce blood lipid levels in mice [30], but the mechanism of lipid lowering by walnut peptides has not been investigated. Accordingly, the purpose of this study was to investigate the effects of walnut meal peptides on body fat distribution and lipid metabolism in a rat model of diet-induced obesity and hyperlipidemia, so as to provide a theoretical basis for the development of functional foods to prevent hyperlipidemia.

## 2. Materials and Methods

### 2.1. Walnut Meal Protein Isolate Preparation

The peeling of walnut kernel was slightly modified according to the method of Mao [31]. Soaked the walnuts in water overnight, cooked them with 2% NaOH at 95 °C for 3 min, then rinsed with water and peeled off. Walnut meal obtained from peeled walnut kernel removed by cold pressing with an oil press. Then the walnut meal was crushed and the remaining walnut oil was removed by soxhlet extraction. Walnut fat-removing meal was first washed with 95% alcohol, then filtered and evaporated the solvent. Dissolved the walnut meal with water at a ratio of 1:16, adjusted the pH to 11 with 2 mol/L NaOH solution and placed it in a 53 °C water bath for extraction for 1.5 h. The supernatant was obtained after centrifugation (4000 r/min, 15 min), and the pH of the supernatant was adjusted to 4.5 with 0.5 mol/L HCl. After standing for 1 h, the solution was centrifuged at 4000 r/min for 15 min to obtain precipitation. Then the pH of the precipitate to neutral was adjusted with 0.5 mol/L NaOH solution, and the precipitation was freeze-dried to form walnut protein isolate.

### 2.2. Screening of Protease for Polypeptide Hydrolysis of Walnut Meal

The walnut protein separation powder was hydrolyzed with basic protease, neutral protease, papain, trypsin, pepsin and alcalase 2.4L respectively under the optimal enzymatic hydrolysis conditions as shown in Table A1. The best enzyme was selected by measuring the inhibition of pancreatic lipase and the inhibition of cholesterol solubility of different hydrolysates.

### 2.3. In Vitro Evaluation of Hypolipidemic Activity of Peptides

#### 2.3.1. Inhibition of Cholesterol Micellar Solubility

The cholesterol-binding capacity of walnut peptides in vitro was determined according to the method of Nagaoka et al. [32] with slight modification. Briefly, a solution (1 mL) containing 10 mM sodium taurocholate, 0.4 mM cholesterol, 1 mM oleic acid, 132 mM NaCl, 5 mg/mL walnut peptides and 15 mM sodium phosphate buffer (pH 7.4), was prepared by ultrasonic treatment (power: 400 W, frequency: 20 kHz, 20 min). This solution was incubated at 37 °C for 24 h, and then centrifuged at 8000 g for 30 min to obtain the supernatant. Finally, the cholesterol solubility reduction rate of the supernatant was measured with a cholesterol kit. The micellar solubility reduction of cholesterol was calculated according to the following equation:

Cholesterol solubility reduction (%) = (Co-Cs)/Co × 100(1)

where Co is the concentration of cholesterol in micellar solution without peptides, and Cs is the concentration of cholesterol in micelles when peptides are added.

#### 2.3.2. Pancreatic Lipase Inhibition in Vitro Assay

The pancreatic lipase inhibition rate was determined according to the method proposed by Prados et al. [33] with some modifications. Dissolved p-nitrophenyl acetate in DMSO to prepare a 50 mM stock solution, then diluted to 10 mM with distilled water. Porcine pancreatic lipase was dissolved in water to prepare a solution with a concentration of 10 mg/mL, and then centrifuged at 13,000 rpm for 5 min. The total volume of the entire reaction system is 0.2 mL, and the composition was as follows: 0.02 mL extract, 0.02 mL enzyme solution, 0.14 mL Tris buffer (pH 7.4), 0.02 mL substrate solution. The absorbance valued of the released p-nitrophenol was measured at 405 nm. The inhibition rate of pancreatic lipase was calculated according to the following formula:

Inhibition of pancreatic lipase (%) = (Amax-(As-Aso))/Amax × 100(2)

where Amax is the absorbance of the solution without any peptide (the maximum activity of the enzyme), A_S_ is the absorbance of the enzyme-containing solution after adding peptides, and A_So_ is the absorbance of sample solution without enzyme.

### 2.4. Analysis of Amino Acid Composition

Walnut protein isolate and walnut meal peptides were dissolved with 6 mol/L of HCl (110 °C, 22 h) respectively, and the amino acid composition of the hydrolysates were analyzed by an amino acid analyzer (HP1100, Agilent) after passing through a 0.45 μm inorganic filter membrane. Identification of amino acids was performed by comparing the retention time of the sample with those of the standards.

### 2.5. Animal and Experimental Design

Thirty 6-week-old, specific pathogen-free Sprague Dawley (SD) rats (male, weighing 180 ± 20 g) obtained from the Experimental Animal Center of the Fourth Military Medical University (Xi’an, China), were kept in an animal room under a well-maintained and hygienic environment (controlled temperature: 25 ± 1 °C, humidity: 55 ± 5%, and the day and night were respectively 12 h of circulation) with free access to the assigned diets and sterile water. After a 7-day acclimation period, all rats were randomly divided into five treatment groups: ND group (rats fed normal diet, *n* = 6), HFD group (rats fed high-fat diet, *n* = 6), LWMP (rats fed low dose walnut meal peptides 200 mg/kg, *n* = 6), MWMP (rats fed middle dose walnut meal peptides 400 mg/kg, *n* = 6), and HWMP (rats fed high dose walnut meal peptides 800 mg/kg, *n* = 6). The composition of normal feed and high-fat feed is shown in Table A2. After 4 weeks of continuous experimental administration, the rats were anesthetized with 1 mg/kg pentobarbital sodium to obtain blood and tissues. All experimental protocols were carried out in accordance with the guidelines of National Institutes of Health on the care and use of laboratory animals (NIH Publications No. 8023, revised 1978).

### 2.6. Histological Analysis

Fat bodies around the epididymis, liver and cecum tissues were collected and fixed in a 4% paraformaldehyde solution, and then stained with hematoxylin and eosin (H&E) for pathological examination [34]. For the oil red O staining of liver tissue, section samples were processed and sectioned sliced (4 μm) under freezing conditions and stained with oil red O solution. Tissues were washed briefly in 60% isopropanol, and then rinsed with distilled water. Finally, the observation and photographing of the stained sections were performed with a microscope (Olympus, Tokyo, Japan) equipped with a digital camera (Olympus, Tokyo, Japan).

### 2.7. Biochemical Analysis

#### 2.7.1. Serological Analysis

The blood samples were collected in non-heparinized vacuum tubes, and then centrifuged at 4000 rpm for 10 min at 4 °C to collect the serum. The concentrations of total triglyceride (TG), total cholesterol (TC), low-density lipoprotein cholesterol (LDL-C), high-density lipoprotein cholesterol (HDL-C), alanine aminotransferase (ALT), aspartate aminotransferase (AST), apolipoprotein-B (Apo-B) and apolipoprotein-A1 (Apo-A1) were measured with a microplate reader by using commercial kits obtained from Nanjing Jiancheng Bioengineering Institute (Nanjing, China). The atherogenic index (AI) of serum was calculated according to the following equation:

AI = (TC − HDL-C)/HDL-C(3)

#### 2.7.2. Liver Biochemical Analysis

Frozen rat liver tissues were homogenized in normal saline (w:v = 1:9), and then centrifuged (3000 g, 10 min) to obtain the supernatant. The content of TG and TC and the protein concentration in the supernatant of liver homogenate were determined by using commercial kits obtained from Nanjing Jiancheng Bioengineering Institute (Nanjing, China).

### 2.8. Real-Time Quantitative PCR

Total RNA was isolated from the liver by using the Trizol reagent and reverse-transcribed into cDNA using a HiFiScript cDNA Synthesis Kit (Cwbio, Beijing, China) and real time PCR was performed using an UltraSYBR Mixture (Cwbio, Beijing, China). The cycle conditions were as follows: 10 min at 95 °C followed by 40 cycles of incubation at 95 °C for 5 s, then 57.8 °C for 20 s, and 72 °C for 40 s. The primer sequences of fatty acid synthase (FAS), HMG-CoA reductase (HMGR), Lecithin cholesterol acyl-transferase (LCAT) and cholesterol 7α-hydroxylase (CYP7A1) are listed in Table A3.

### 2.9. Statistical Analysis

All experiments were carried out in triplicate and the continuous variables were expressed as means ± SD (standard deviation). Data were analyzed by one-way analysis of variance (ANOVA) using the SPSS package software (SPSS 17.0 for Windows, SPSS Inc., Chicago, IL, USA). For all analyses, the *p*-value of 0.05 or less was considered statistically significant.

## 3. Results

### 3.1. Screening of Protease for Polypeptide Hydrolysis of Walnut Meal

**Figure 1:**
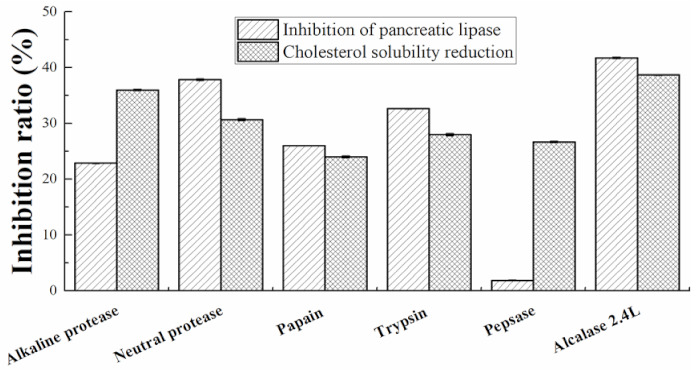
Comparison of the effect of different proteases on in vitro solubilization of cholesterol in mixed micelles and inhibition of pancreatic lipase.

There are obvious differences in the in vitro hypolipidemic activity of different proteolysis products, which may be caused by the different hydrolysis sites of different enzymes. As shown in Figure 1, the enzymatic hydrolysis product of alcalase 2.4L has a higher potency to impair the solubilization of cholesterol in mixed micelles and to inhibit pancreatic lipase than the other five enzymes. Under the optimal conditions of alcalase 2.4L: pH 7.9, hydrolysis temperature: 62.4 °C, enzyme content: 6.98%, substrate concentration: 4.13%, and hydrolysis time: 4.23 h. Walnut meal protein powder was hydrolyzed to obtain walnut meal peptide. The protein content was 93%, which was determined by the method of Lowry [35].

### 3.2. Amino Acid Composition of WMP

The amino acid composition of the protein extract will determine its effect on serum cholesterol levels. The cholesterol-lowering effect will increase as the ratio of lysine/arginine decreases [36]. The amino acid analysis results of walnut protein isolate and walnut meal peptides are shown in Table 1. Compared with the amino acid composition of walnut protein isolate, the hydrophobic amino acid content of WMP increased significantly. WMP was rich in Glu, Arg, Asp and Leu, which accounted for 20.04 mg/100 g, 15.52 mg/100 g, 12.21 mg/100 g and 6.67 mg/100 g, respectively (Table 1).

### 3.3. Effect of WMP on the Body Weight and Tissue Weight of Rats

As shown in Table 2, after 4 weeks of feeding, the final body weight, liver weight and adipose tissue weight of the rats in the HFD group were dramatically increased compared to the ND group (*p* < 0.05). However, these abnormal increases were effectively inhibited by the administration of walnut meal peptides. Moreover, the total energy intake of the rats fed with high-fat diet was obviously higher than that of rats fed with normal diet, which resulted in an abnormal increase in the weight of rats. Interestingly, the total energy intake of rats in the walnut meal peptides treatment group was close to that of the HFD group, but the weight of the rats in the walnut meal peptides treatment group was markedly lower than that of the HFD group, suggesting that walnut meal polypeptide has potential anti-obesity benefits.

### 3.4. Antihyperlipidemic Effect of WMP on Hyperlipidemic Rats

**Figure 2:**
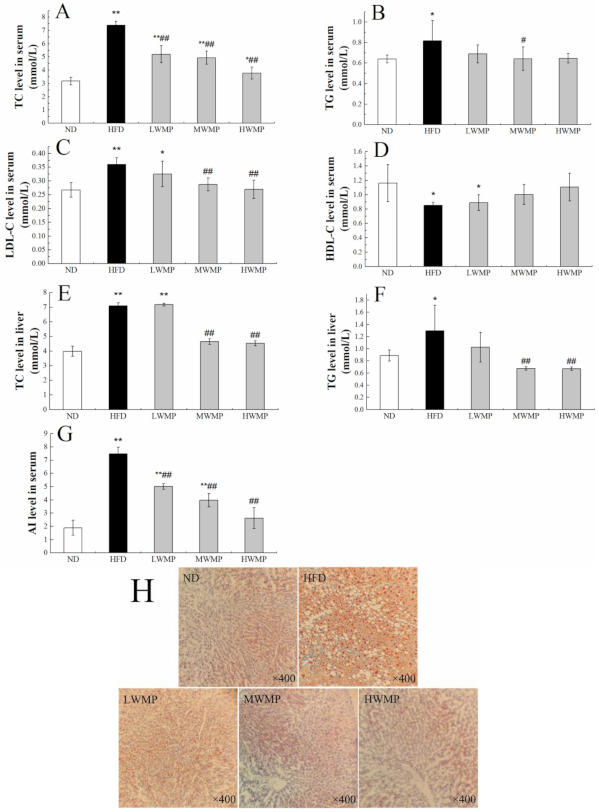
The regulating effect of WMP on the balance of lipid metabolism in high-fat diet rats. (**A**) Serum TC level, (**B**) serum TG level, (**C**) serum LDL-C level, (**D**) serum HDL-C level, (**E**) liver TC level, (**F**) liver TG level, (**G**) serum AI level and (**H**) representative image of Oil Red O staining of liver tissue. ND, HFD, LWMP, MWMP and HWMP denote the group of rats fed with normal diet, high-fat diet, low dose walnut meal peptides, middle dose walnut meal peptides and high dose walnut meal peptides, respectively. Data are expressed as mean ± SD (n = 6). * and ** represent *p* < 0.05 and *p* < 0.01 compared to normal group, # and ## indicate *p* < 0.05 and *p* < 0.01 compared to model group, respectively.

The improvement effect of WMP on hyperlipidemia induced by high-fat diet in rats was further investigated. As shown in Figure 2, the serum total cholesterol (TC), total triglyceride (TG) and low-density lipoprotein cholesterol (LDL-C) levels in rats (Figure 2) was obviously increased by HFD, and these abnormal increases were ameliorated by WMP treatment. Meanwhile, HFD also markedly increased the hepatic TC, TG levels in rats (Figure 2E,F). Serum atherogenic index (AI) values were significantly different among each group. As expected, the administration of WMP effectively inhibited abnormal changes in these parameters (Figure 2G). These results indicate that WMP administration plays a critical role in preventing hyperlipidemia. Meanwhile, rats fed the high-fat diets had an obvious increase in the number of oil droplets in the liver cells compared with rats fed the normal diet and walnut meal peptides (Figure 2H). Compared with the HFD group, the WMP groups had normal cell structure and significantly fewer lipid droplets (Figure 2H). Collectively, these results indicated that WMP could improve lipid metabolism and alleviate the hepatic steatosis in HFD-fed rats.

### 3.5. Effects of WMP on Apolipoproteins

**Figure 3:**
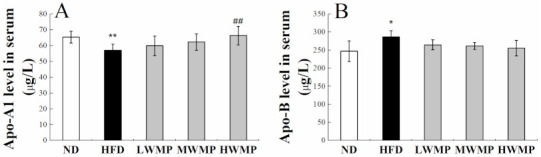
Effect of WMP on serum Apo-A1 (**A**) and Apo-B level (**B**) of high-fat diet rats. ND, HFD, LWMP, MWMP and HWMP denote the group of rats fed with normal diet, high-fat diet, low dose walnut meal peptides, middle dose walnut meal peptides and high dose walnut meal peptides, respectively. Data are expressed as mean ± SD (*n* = 6). * and ** represent *p* < 0.05 and *p* < 0.01 compared to normal group, ## indicate *p* < 0.01 compared to model group, respectively.

Apolipoprotein A1(Apo-A1) is a structural protein of high-density lipoprotein (HDL) that plays a critical role in cardiovascular protection [37,38], which mainly affects the reverse cholesterol transport process of HDL [39]. Apolipoprotein B (Apo-B) is the most important element in very low-density lipoprotein (VLDL) assembly [40]. As exhibited in Figure 3, Apo-A1 activities in the serum of model rats were markedly lowered by 12.74% compared with the normal rats (*p* < 0.01) while the activity of Apo-B in the serum of model rats was 15.83% higher than that of normal rats (*p* < 0.05), indicating that high-fat feeding resulted in a negative effect on lipid metabolism homeostasis in rats. The supplementation of walnut meal peptides shows a significant impact for Apo-A1 and Apo-B in HFD-fed rats. These results demonstrated that WMP could regulate the lipid metabolism process.

### 3.6. Effects of WMP on the Genes involved in Enzymes Related to Cholesterol Metabolism in Liver

**Figure 4:**
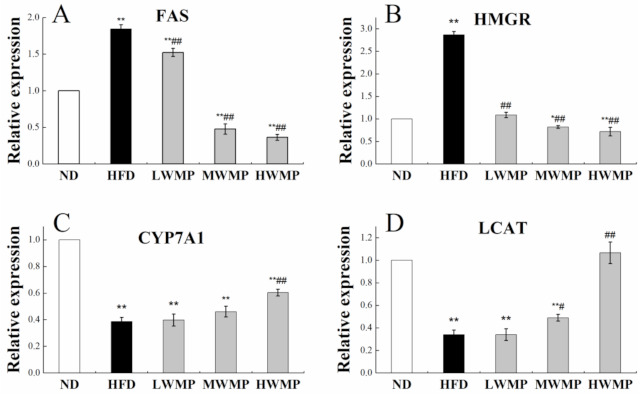
Effect of WMP on the expression of FAS (**A**), HMGR (**B**), CYP7A1 (**C**) and LCAT (**D**) in liver of high-fat diet rats. ND, HFD, LWMP, MWMP and HWMP denote the group of rats fed with normal diet, high-fat diet, low dose walnut meal peptides, middle dose walnut meal peptides and high dose walnut meal peptides, respectively. Data are expressed as mean ± SD (*n* = 6). * and ** represent *p* < 0.05 and *p* < 0.01 compared to normal group, # and ## indicate *p* < 0.05 and *p* < 0.01 compared to model group, respectively.

We further investigated the effect of walnut meal peptides on the lipid metabolism marker enzymes in HFD-induced obese rats, including fatty acid synthase (FAS), HMG-CoA reductase (HMGR), lecithin cholesterol acyltransferase (LCAT) and cholesterol 7α-hydroxylase (CYP7A1). Thus, RT-qPCR was performed to analyze the hepatic mRNA expression levels of genes involved in the lipid metabolism marker enzymes (Figure 4). Significantly increased relative expression of FAS and HMGR was observed in HFD rats when compared with control rats (*p* < 0.01). However, walnut meal peptides treatment significantly (*p* < 0.05) reduced the expression levels of FAS and HMGR in the liver of high-fat diet rats (Figure 4A,B). Additionally, the relative expression of the hepatic CYP7A1 and LCAT of normal and experimental rats are displayed in Figure 4C,D. The liver of the rats fed HFD showed an obvious decrease (*p* < 0.01) in CYP7A1 and LCAT gene expressions. As expected, the administration of walnut meal peptides to obese rats effectively restored the expression of LCAT and CYP7A1.

### 3.7. Effects of WMP on HFD-Induced Hepatic Damage

**Figure 5:**
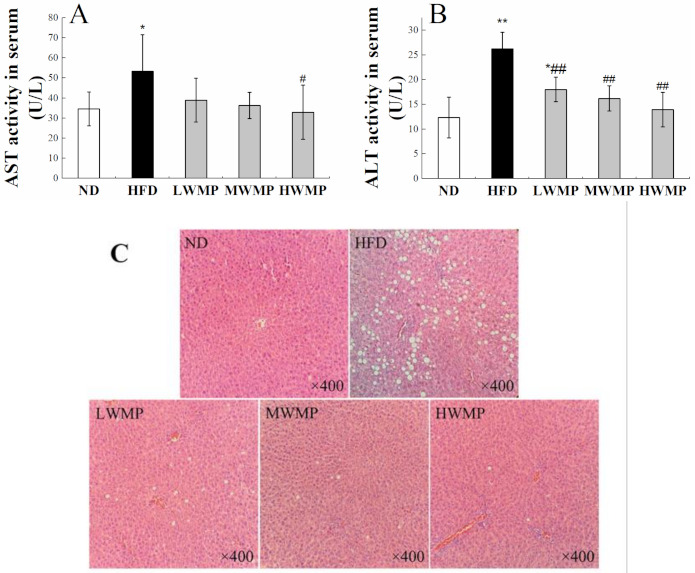
Supplementary effects of the walnut peptides on AST (**A**), ALT activity (**B**), H&E (**C**) staining of hepatic tissue in rats fed a high-fat diet. ND, HFD, LWMP, MWMP and HWMP denote the group of rats fed with normal diet, high-fat diet, low dose walnut meal peptides, middle dose walnut meal peptides and high dose walnut meal peptides, respectively. Data are expressed as mean ± SD (*n* = 6). * and ** represent *p* < 0.05 and *p* < 0.01 compared to normal group, # and ## indicate *p* < 0.05 and *p* < 0.01 compared to model group, respectively. Original magnification ×400.

The activity of serum transaminase is a sensitive and reliable indicator of liver function [41]. Studies have shown that inflammation, necrosis, poisoning and injury of liver cells can cause alanine aminotransferase (ALT) and aspartate aminotransferase (AST) to penetrate into the blood, leading to increase in serum ALT and AST levels [42]. As shown in Figure 5A,B, the serum AST, ALT levels of the rats in HFD group were significantly increased compared with the ND group (*p* < 0.05), and WMP administration effectively reduced these abnormal increases.

Histological analyses of the liver section stained by H&E further confirm the aforementioned results. As displayed in Figure 5C, consumption of HFD for four weeks caused a severe injury to the rat liver, showing characteristics such as cytoplasmic vacuolation, degeneration, inflammatory cell infiltration, and loss of cell boundaries. As expected, the liver cells of rats treated with WMP showed a similar appearance to normal hepatocytes, with obvious cell boundaries, intact cytoplasm and clear nucleus (Figure 5C). The phenomenon of cytoplasmic vacuolation has been significantly improved. These results indicate that WMP may have a significant effect in alleviating liver damage caused by HFD.

### 3.8. Effects of WMP on the Hematoxylin-Eosin (H&E) Staining of Cecum Tissue and Fat Bodies Around the Epididymis

**Figure 6:**
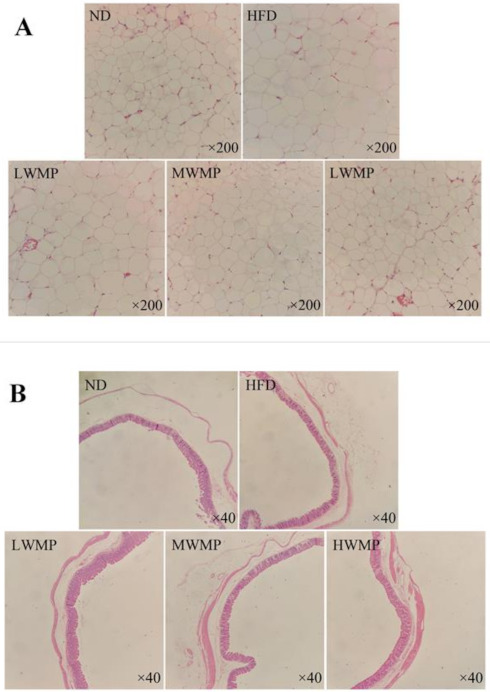
Supplementary effects of the walnut meal peptides on epididymal adipocyte morphologies (**A**) and cecum tissue (**B**) in rats fed a high-fat diet. ND, HFD, LWMP, MWMP and HWMP denote the group of rats fed with normal diet, high-fat diet, low dose walnut meal peptides, middle dose walnut meal peptides and high dose walnut meal peptides, respectively. Original magnification ×200 and ×40.

The H&E stained epididymal adipose tissue sections of rats in each group are shown in Figure 6A. The diameter of epididymal fat cells in rats on a high-fat diet for four consecutive weeks is larger than that of normal rats, and WMP supplementation effectively reduced the average size of epididymal fat cells, and the fat cells are arranged more closely. The morphology of adipocytes in the HWMP group was similar to that of the normal group. Furthermore, the microscopic analysis of rat cecum slices is shown in Figure 6B. The length of cecal villi of rats in the high-fat diet group was obviously reduced compared with that of normal rats, showing an abnormal cecal wall. However, WMP intervention effectively increased the villus length and mucosal thickness of the cecum, indicating that WMP may have a positive effect on intestinal health.

## 4. Discussion

Plant-derived peptides have been proven to possess a variety of biological activities including hypolipidemic activity [17]. Some acidic amino acids such as Asp and Glu [43], hydrophobic amino acids such as Ile, Leu, Val [44,45] and His [46], all show strong antioxidant properties and contribute to lipid reduction. Arginine supplementation can reverse vascular endothelial dysfunction induced by high-fat diet [47]. Compared with soy protein, which is known to have good cholesterol-lowering activity, the Lys/Arg value of walnut protein is significantly lower [19] (Table 1). Therefore, the hypolipidemic activity of walnut meal peptides may be closely related to the composition of amino acid and hydrophobicity. The pancreatic lipase enzyme is a crucial enzyme for the hydrolysis of dietary fat. Inhibition of pancreatic lipase enzyme, which hydrolyzes triglycerides into mono triglycerides and free fatty acids, can reduce fat absorption [48]. The inhibition of the micellar solubilization of cholesterol would induce cholesterol malabsorption in the jejunum, as has been shown for peptides derived from bovine beta-lactoglobulin [31], ultimately reducing serum cholesterol. In vitro experiments demonstrated that walnut meal peptides interfered with the micellar solubility of cholesterol and the activity of pancreatic lipase, indicating possible mechanisms for their hypocholesterolemic and anti-obesity effects.

Hyperlipidemia, as a metabolic syndrome, is due to a lipid metabolism disorder in the body. High-fat diet feeding in rats induces metabolic syndrome and nonalcoholic fatty liver disease [49]. Our results indicated that a high-fat model was successfully established by feeding SD rats with a high-fat diet for four consecutive weeks. In the present study, the increase in serum and liver lipid levels in rats fed a high-fat diet was consistent with other studies [8,50,51]. However, the serum and liver lipid levels of HFD rats supplemented with walnut meal peptides were restored to levels close to those of ND rats. Meanwhile, the liver is an essential organ for fat metabolism, and lipid droplets are usually accumulated in liver cells when there is excess body fat, causing fatty liver and liver damage. In our study, the liver of HFD-fed rats showed severe lipid accumulation and abnormal liver index, which was consistent with previous report [52]. The administration of WMP reduced the concentrations of ALT and AST in the serum of HFD-fed rats compared with the normal rats (Figure 5A,B), and alleviated liver fat accumulation and hepatic tissue damage (Figure 2H and Figure 5C). These results indicate that the intervention with WMP can effectively protect the liver of rats fed a high-fat diet from fat damage.

In the pathway of bile acid biosynthetic production, CYP7A1 acts as a rate-limiting enzyme [53]. HMGR, the rate-limiting enzyme for the biosynthesis of plasma cholesterol, plays a vital role in the regulation of lipid metabolism [54]. LCAT acts as a key participant in the process of reverse cholesterol transport. Lack or inactivity of LCAT increases the risk of atherosclerotic disease [55]. FAS is a lipogenic enzyme involved in the energy metabolism of synthesis of long-chain fatty acids, which is reported as a potential therapeutic target for obesity and cancer [56]. In this study, increases in the expression of FAS and HMGR and reduction in CYP7A1 and LCAT expression were observed in the liver of high fat diet rats, in line with findings of previous studies using the same rat model [57,58]. However, the intervention of WMP inhibited these trends (Figure 4). However, there is no significant increase in CYP7A1, which indicates that WMP may has limited effect on, regulate the catabolism of cholesterol by stimulating the conversion of cholesterol into bile acid. In summary, WMP may achieve the effect of hypolipidemic by inhibiting the synthesis of cholesterol and fatty acids.

Previous research demonstrated that there is a close relationship between a high-fat diet and gut health [59]. As reported by Attene-Ramos et al. [60], the byproducts of lipid metabolism in a high-fat diet would cause an injury in the mucosa of the large intestine and suppress the growth of muscle cells. The intervention of WMP increased mucosal thickness of the cecum, which is important for preventing the invasion of harmful compounds and pathogenic microorganisms and maintaining the stability of the intestinal environment. Moreover, WMP also increases the length of the cecum villus (Figure 6B). The increase in the length of the villus would increase the absorption area of the intestines, and promote the full absorption and utilization of the body’s needed nutrients [61]. In addition, studies have shown that HFD may induce hyperlipidemia by affecting the composition of the gut microbiota, achieved by changing the relativity abundance of pro-inflammatory and pathogenic bacteria [62]. Therefore, we can further explore the hypolipidemic effect of WMP by studying the changes of intestinal flora. 

In the current study, we have studied the beneficial effects of walnut meal peptides on rats on long-term high-fat diet. Our results suggest that the oral administration of WMP for four weeks counteracted the weight gain and increases in serum liver enzymes and serum and liver cholesterol and TG content that takes place in rats fed an HFD. Specifically, the intake of walnut meal peptides improved liver damage and abnormal levels of transaminases, i.e., AST and ALT. Our data also suggests that WMP treatment restores the disordered lipid metabolism caused by an HFD, which was probably achieved by regulating the activity of lipid metabolism related enzymes and the redistribution of cholesterol in the blood and liver [63]. In clinical studies, regular nut consumption has a dose-related cholesterol lowering effect, thus small amounts only have a modest effect [26]. Due to their high activity, WMP may be used at doses smaller than when using whole walnuts to achieve a cholesterol lowering effect. The current work contributes to the development of WMP as a promising functional food for dietary strategies to prevent abnormal lipid metabolism.

## Figures and Tables

**Table 1:** Amino acid composition of walnut meal protein and walnut meal peptides.

Amino Acid	Hydrophobicity (kJ /mol)	Walnut Protein Isolate (mg/100 g)	Walnut Meal Peptides (mg/100 g)
Asp	2.25	9.43 ± 0.07 ^a^	12.21 ± 0.09 ^b^
Thr	1.85	3.58 ± 0.10 ^a^	4.36 ± 0.12 ^b^
Ser	0.17	4.39 ± 0.05 ^a^	5.49 ± 0.06 ^b^
Glu	2.30	17.36 ± 0.35 ^a^	20.04 ± 0.36 ^b^
Gly	0	3.83 ± 0.14 ^a^	3.95 ± 0.15 ^a^
Ala	3.10	7.53 ± 0.09 ^a^	8.43 ± 0.10 ^b^
Cys	4.20	0.00 ± 0.00 ^a^	0.00 ± 0.00 ^a^
Val	7.05	4.99 ± 0.01 ^a^	5.47 ± 0.01 ^b^
Met	5.45	2.27 ± 0.11 ^a^	2.13 ± 0.10 ^a^
Ile	12.40	4.29 ± 0.05 ^a^	3.78 ± 0.04 ^b^
Leu	10.10	5.81 ± 0.15 ^a^	6.67 ± 0.19 ^b^
Tyr	12.00	3.52 ± 0.13 ^a^	3.80 ± 0.11 ^a^
Phe	11.10	3.55 ± 0.03 ^a^	3.32 ± 0.04 ^a^
Lys	6.25	3.06 ± 0.05 ^a^	2.17 ± 0.03 ^b^
His	2.10	2.36 ± 0.08 ^a^	2.26 ± 0.07 ^a^
Arg	3.10	11.18 ± 0.03 ^a^	15.52 ± 0.02 ^b^
Pro	10.85	1.73 ± 0.13 ^a^	1.92 ± 0.17 ^a^
Lys/Arg		0.27	0.14

Data are presented as mean ± SD (*n* = 3). Values denoted by different letters differ significantly (*p* < 0.05) among one another.

**Table 2:** Effects of body weights, total energy intake and tissue weights in rats fed different diets for four consecutive weeks.

Parameters	ND	HFD	LWMP	MWMP	HWMP
Initial body weight (g)	273.92 ± 5.53 ^a^	282.29 ± 3.77 ^a^	282.22 ± 3.56 ^a^	281.00 ± 7.79 ^a^	279.50 ± 5.30 ^a^
Final body weight (g)	358.74 ± 12.74 ^a^	429.25 ± 10.40 ^b^	402.26 ± 14.08 ^b^	389.90 ± 10.25 ^a^	387.77 ± 7.11 ^a^
Body weight gain (g)	89.24 ± 9.28 ^a^	151.08 ± 13.56 ^b^	118.12 ± 12.94 ^c^	108.90 ± 2.94 ^a,c^	113.44 ± 4.72 ^a,c^
Fasting body weight (g)	345.92 ± 12.63 ^a^	410.98 ± 16.19 ^b^	374.23 ± 13.01 ^a^	368.17 ± 9.24 ^a^	363.32 ± 6.74 ^a^
Total energy intake(kcal)	8386.24 ± 952.24 ^a^	9016.60 ± 1061.88 ^b^	8883.24 ± 1047.42 ^b^	8796.61 ± 786.81 ^b^	8712.66 ± 507.61 ^b^
Liver weight (g)	7.72 ± 0.40 ^a^	9.13 ± 0.34 ^b^	9.06 ± 0.45 ^b^	8.83 ± 0.24 ^b^	8.68 ± 0.16 ^a,b^
Epididymis fat (g)	2.04 ± 0.36 ^a^	3.76 ± 0.78 ^b^	3.15 ± 0.79 ^a^	2.79 ± 0.39 ^a^	2.67 ± 0.36 ^a^

ND, HFD, LWMP, MWMP and HWMP denote the group of rats fed with normal diet, high-fat diet, low dose walnut meal peptides, middle dose walnut meal peptides and high dose walnut meal peptides, respectively. Data are presented as mean ± SD (*n* = 6). Values denoted by different letters differ significantly (*p* < 0.05) among one another.

## Data Availability

All data presented in this study are available on request from the corresponding author. The data are not uploaded in publicly accessible databases.

## Appendix A

**Table A1:** The optimum reaction conditions of six proteases.

Types of Proteases	Substrate Concentration	Volume of Enzyme (U/g)	Temperature (°C)	pH
Basic protease	3%	5000	55	8.0
Neutral protease	3%	5000	45	7.0
Papain	3%	5000	65	6.5
Trypsin	3%	5000	55	7.5
Pepsin	3%	5000	60	3.0
Alcalase 2.4L	3%	5000	62.4	7.7

**Table A2:** Composition of normal feed and high-fat feed.

Composition	Normal Feed (g%)	High Fat Feed (g%)
Corn flour	40 26 10 10 10 2 1 1	20
Wheat flour		13
Bran		5
Fish meal		5
Bean cake		5
Mineral		1
Coarse		0.5
Vitamin complex		0.5
Lard		18
Sucrose		10
Whole milk powder		10
Casein		8
Animal feed premix		2
Calcium bicarbonate		2

**Table A3:** Primer sequence.

Gene	Forward 5′-3′ Primer Sequence	Reverse 5′-3′ Primer Sequence
FAS	TTGAAGAGGAGCGTTCGTGA	GGTTGACAGCAAAATGGGC
HMGR	AGCGTGGTGTGTCTATTCG	GAGACGGATGTAGAGGTTGC
CYP7A1	AGAGAATCATTAGCCGTGCCAG	TAGGGAGACATTTGAGTGAGCGA
LCAT	TGTGCTACCGAAAGACAGAGG	CTTGCCAAAGCCAGGGAC
GAPDH	ACAGCAACAGGGTAATGGAC	TTTGAGGGTGCAGCGAACTT
